# Performance of immune-based and microbiological tests in children with tuberculosis meningitis in Europe: a multicentre Paediatric Tuberculosis Network European Trials Group (ptbnet) study

**DOI:** 10.1183/13993003.02004-2019

**Published:** 2020-07-02

**Authors:** Robindra Basu Roy, Stephanie Thee, Daniel Blázquez-Gamero, Lola Falcón-Neyra, Olaf Neth, Antoni Noguera-Julian, Cristina Lillo, Luisa Galli, Elisabetta Venturini, Danilo Buonsenso, Florian Götzinger, Nuria Martinez-Alier, Svetlana Velizarova, Folke Brinkmann, Steven B. Welch, Maria Tsolia, Begoña Santiago-Garcia, Renate Krüger, Marc Tebruegge

**Affiliations:** 1 Clinical Research Dept, London School of Hygiene and Tropical Medicine, London, UK; 2 Dept of Pediatric Pneumology, Immunology and Intensive Care, Charité–Universitätsmedizin Berlin, Berlin, Germany; 3 Paediatric Infectious Diseases Unit, Hospital Universitario 12 de Octubre, Universidad Complutense de Madrid, Instituto de Investigación Hospital Universitario 12 de Octubre (imas12), RITIP, Madrid, Spain; 4 Paediatric Infectious Diseases, Rheumatology and Immunology Unit, Hospital Universitario Virgen del Rocío, Institute of Biomedicine, Seville, Spain; 5 Malalties Infeccioses i Resposta Inflamatòria Sistèmica en Pediatria, Institut de Recerca Pediàtrica; Hospital Sant Joan de Déu, Barcelona, Spain; 6 Departament de Pediatria, Universitat de Barcelona, Barcelona, Spain; 7 CIBER de Epidemiología y Salud Pública, CIBERESP, Madrid, Spain; 8 Red de Investigación Translacional en Infectología Pediátrica, RITIP, Madrid, Spain; 9 Dept of Health Sciences, University of Florence, Florence, Italy; 10 Paediatric Infectious Disease Unit, Meyer Children's University Hospital, Florence, Italy; 11 Dept of Woman and Child Health and Public Health, Fondazione Policlinico Universitario A. Gemelli IRCCS, Rome, Italy; 12 Dept of Paediatrics and Adolescent Medicine, Wilhelminenspital, Vienna, Austria; 13 Dept of Paediatric Infectious Diseases and Immunology, Evelina London Children's Hospital, Guy's and St. Thomas’ NHS Foundation Trust, London, UK; 14 Dept of Pulmonary Diseases, Medical University, Hospital for Lung Diseases ‘St. Sofia’, Sofia, Bulgaria; 15 Dept of Paediatric Pulmonology, Ruhr University Bochum, Bochum, Germany; 16 Birmingham Chest Clinic and Heartlands Hospital, University Hospitals Birmingham, Birmingham, UK; 17 Second Dept of Paediatrics, National and Kapodistrian University of Athens, School of Medicine, P. and A. Kyriakou Children's Hospital, Athens, Greece; 18 Dept of Paediatric Infectious Diseases, Hospital General Universitario Gregorio Marañón, Madrid, Spain; 19 Dept of Paediatrics, Royal Children's Hospital Melbourne, University of Melbourne, Melbourne, Australia; 20 Dept of Infection, Immunity and Inflammation, UCL Great Ormond Street Institute of Child Health, University College London, London, UK; 21 Joint first authors; 22 A list of the tbnet TB Meningitis Study Group Collaborators can be found in the acknowledgements section

## Abstract

**Introduction:**

Tuberculous meningitis (TBM) is often diagnostically challenging. Only limited data exist on the performance of interferon-γ release assays (IGRA) and molecular assays in children with TBM in routine clinical practice, particularly in the European setting.

**Methods:**

Multicentre, retrospective study involving 27 healthcare institutions providing care for children with tuberculosis (TB) in nine European countries.

**Results:**

Of 118 children included, 54 (45.8%) had definite, 38 (32.2%) probable and 26 (22.0%) possible TBM; 39 (33.1%) had TBM grade 1, 68 (57.6%) grade 2 and 11 (9.3%) grade 3. Of 108 patients who underwent cranial imaging 90 (83.3%) had at least one abnormal finding consistent with TBM. At the 5-mm cut-off the tuberculin skin test had a sensitivity of 61.9% (95% CI 51.2–71.6%) and at the 10-mm cut-off 50.0% (95% CI 40.0–60.0%). The test sensitivities of QuantiFERON-TB and T-SPOT.TB assays were 71.7% (95% CI 58.4–82.1%) and 82.5% (95% CI 58.2–94.6%), respectively (p=0.53). Indeterminate results were common, occurring in 17.0% of QuantiFERON-TB assays performed. Cerebrospinal fluid (CSF) cultures were positive in 50.0% (95% CI 40.1–59.9%) of cases, and CSF PCR in 34.8% (95% CI 22.9–43.7%). In the subgroup of children who underwent tuberculin skin test, IGRA, CSF culture and CSF PCR simultaneously, 84.4% had at least one positive test result (95% CI 67.8%–93.6%).

**Conclusions:**

Existing immunological and microbiological TB tests have suboptimal sensitivity in children with TBM, with each test producing false-negative results in a substantial proportion of patients. Combining immune-based tests with CSF culture and CSF PCR results in considerably higher positive diagnostic yields, and should therefore be standard clinical practice in high-resource settings.

## Abstract 2

**All existing immunological and microbiological TB tests have suboptimal sensitivity in children with TBM. Combining immune-based tests with CSF culture and PCR results in far higher positive diagnostic yields, and should therefore be standard practice.** http://bit.ly/2TSAArl

## Introduction

Globally, an estimated 1 million children and adolescents develop active tuberculosis (TB) annually, with the majority of the disease burden occurring in low-resource countries [1]. In most European countries the incidence of TB disease has been declining steadily over recent decades, but drug-resistant *Mycobacterium tuberculosis* has brought new challenges, particularly in Eastern Europe [2].

TB meningitis (TBM) is an uncommon manifestation of TB disease, but is associated with significant morbidity and mortality, even in high-resource settings [3]. Children with TBM frequently present with nonspecific symptoms and without a history of TB contact, and diagnosing TBM is therefore often challenging [4–6]. Importantly, previous data suggest that delays in diagnosis are linked to poor outcomes [7].

TB diagnostics used in routine clinical practice have evolved significantly over the past two decades, with the introduction of interferon-γ release assays (IGRAs) and a variety of commercial molecular assays [8]. Recent data show that both IGRAs and molecular TB assays are widely available across Europe, and are used extensively by paediatric TB specialists [9, 10].

Immune-based TB tests, comprising tuberculin skin tests (TSTs) and IGRAs, are commonly used as adjunctive diagnostic tools in children with suspected TB disease [8], but the existing data on the performance of IGRAs specifically in children with TBM remain very limited [11, 12]. In addition, although a small number of studies have investigated the use of molecular TB assays in children with suspected TBM, most of these were small, limited to a single study site, or from low-resource settings where late presentations are likely to be more common than in Europe [4, 13, 14]. Furthermore, most studies were conducted under protocolised study conditions, and therefore prone to overestimating test sensitivity compared to performance in routine clinical settings.

This study aimed to determine the sensitivity of immunological, conventional microbiological and molecular TB tests in children with TBM in the context of routine clinical care in Europe. Secondary aims were to describe clinical features and radiological findings at presentation, and to evaluate the Uniform TBM Research Case Definition (UTRCD) score in the European setting.

## Methods

European members of the Paediatric Tuberculosis Network European Trials Group (ptbnet), which at that point in time included 214 clinicians and researchers based in 31 European countries [9, 10, 15, 16], were invited to retrospectively report children and adolescents (aged 0–16 years) with TBM who had received healthcare at their institution. The study opened in February 2016 and reporting closed in August 2016. Data were collected via a web-based tool creating a standardised dataset for each case. The study was reviewed and approved by the human ethics committee of the Charité Universitätsmedizin Berlin and the ptbnet steering committee. No personal or identifiable data were collected during the conduct of this study.

### Classification of cases

Cases were categorised as definite TBM, probable TBM or possible TBM, according to consensus definitions based on the UTRCD score (supplementary table S1) [3], with minor modifications, as follows. The item “history of close contact with an individual with pulmonary TB or a positive TST or IGRA”, scoring 2 points in the original criteria was split into two separate items: 1) history of close contact with an individual with pulmonary TB and 2) a positive TST (≥10 mm) and/or IGRA, each scoring one point if present. Furthermore, “choroidal tubercles” was added to the category “evidence of tuberculosis elsewhere”, scoring 1 point if present; the maximum category score of 4 was retained. If no data were available for one particular item, the respective item was scored as 0. Briefly, definite TBM was defined as a patient with clinical entry criteria (headache, irritability, vomiting, fever, neck stiffness, convulsions, focal neurological deficits, altered consciousness or lethargy) plus one or more of the following: acid-fast bacilli detected in cerebrospinal fluid (CSF), *M. tuberculosis* cultured from CSF or *M. tuberculosis* detected by PCR in CSF. Probable TBM was defined as presence of clinical entry criteria plus a total diagnostic score of ≥12, and possible TBM as presence of clinical entry criteria plus a total diagnostic score of 6–11.

### Disease severity

Disease severity was graded according to the modified British Medical Research Council (BMRC) criteria [17, 18]. In brief, grade 1 corresponds to a Glasgow coma score of 15 with no neurological signs, grade 2 a score of 11–14 or a score of 15 with focal neurological signs, and grade 3 a score of ≤10.

### Statistical analysis

Nonparametric two-tailed Mann–Whitney U-tests were used to compare continuous variables. The sensitivities of diagnostic tests were compared using two-tailed Fisher's exact tests. In cases where a TST was performed and reported as negative, but with no result of induration in millimetres available, it was considered as missing data at the 5-mm threshold and as a negative result at the 10-mm threshold; if a TST was reported as positive without induration size, it was considered as positive at the 10-mm threshold, and hence also positive at the 5-mm threshold. The 95% confidence intervals around proportions were calculated using the Wald method. Multivariate logistic regression analyses were carried out for associations between positive IGRA, positive TST (at the 5-mm and 10-mm thresholds) and indeterminate IGRA as outcome variables, with predictor variables of age, sex, bacille Calmette–Guérin (BCG) vaccination status, TBM staging and definite TBM diagnosis. Models were evaluated using the Hosmer–Lemeshow goodness-of-fit test and receiver operating characteristic curves showed an area under the curve ≥0.7. The primary outcome measures were odds ratios. The 95% confidence interval was calculated for each odds ratio, and p-values <0.05 were considered significant. Analyses were done using Prism (version 8.0; GraphPad, La Jolla, CA, USA) and Stata (version 12.1; StataCorp, College Station, TX, USA). The study is reported in accordance with Standards for Reporting of Diagnostic Accuracy Studies guidelines.

## Results

27 healthcare institutions, situated in Bulgaria (n=1), Finland (n=1), Germany (n=3), Greece (n=1), Italy (n=3), Slovenia (n=1), Spain (n=12), Sweden (n=2) and the United Kingdom (n=3), contributed cases to the study.

118 children were included in the final analysis, comprising 54 (45.8%) definite, 38 (32.2%) probable and 26 (22.0%) possible TBM cases. With regards to disease severity, 39 (33.1%) were BMRC TBM grade 1, 68 (57.6%) grade 2 and 11 (9.3%) grade 3.

The demographic details are shown in table 1. The median (interquartile range (IQR)) age was 2.7 (1.1–6.4) years. Although the majority (89.8%) of children had been born in Europe, almost half of these (48.3%) were from families with one or both parent(s) originating from a country with high TB prevalence. Almost half (41.5%) had a history of TB contact. A test for HIV was performed in 73 (61.9%) children; only two (2.7%) were positive.

**TABLE 1:** Baseline demographic data and clinical features at presentation

**Age years**	2.7 (1.1–6.4)
**Male:female**	1.1:1.0
**Born in Europe**	106 (89.8)
**Born in Europe with one or both parent(s) originating from a high TB incidence country**	57 (48.3)
**Born outside Europe**	12 (10.2)
Africa	6 (50.0)
Asia	3 (25.0)
South America	3 (25.0)
**Prior BCG vaccination**	
Yes	22 (18.6)
No	80 (67.8)
Unknown	16 (13.6)
**Known TB contact**	
Yes	49 (41.5)
No	67 (56.7)
Unknown	2 (1.7)
**Constitutional symptoms at presentation**	
Fever	99 (83.9)
Weight loss	32 (27.1)
Night sweats	10 (8.5)
**Neurological symptoms at presentation**	
Vomiting	70 (59.3)
Altered consciousness	55 (46.6)
Headache	52 (44.1)
Lethargy	27 (22.9)
Cranial nerve palsy	26 (22.0)
Seizures	25 (21.2)
Ataxia	5 (4.2)
Paresis	3 (2.5)

Data are presented as median (interquartile range), n or n (%). TB: tuberculosis; BCG: bacille Calmette–Guérin.

The commonest constitutional symptom at presentation was fever. The most common neurological symptoms at presentation comprised vomiting, headache and altered level of consciousness (table 1).

### Distribution of the UTRCD score among subgroups

**FIGURE 1:**
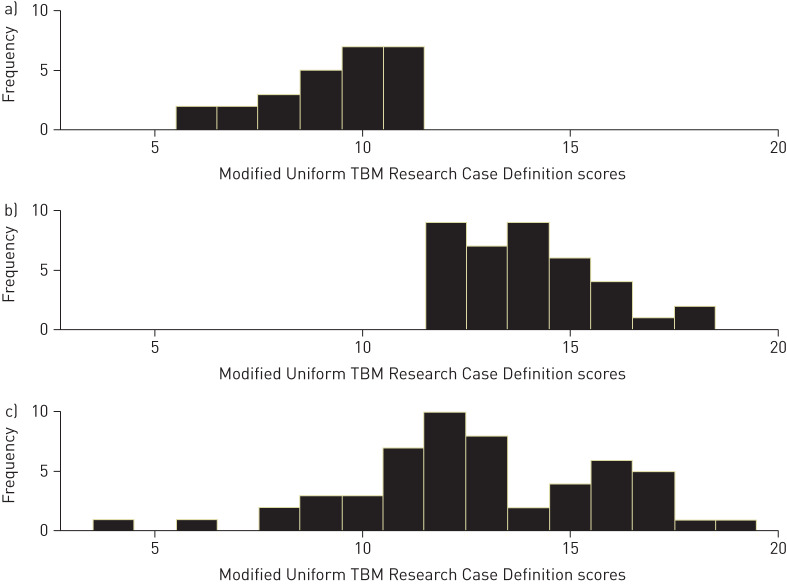
Distribution of modified Uniform Tuberculous Meningitis Research (TBM) Case Definition scores among cases with a) possible, b) probable and c) definite tuberculous meningitis.

In the group of patients with definite TBM the mean±sd score was 12.8±3.1 (range 4–19). In the patients with probable or possible TBM combined, the mean score was 12.1±2.8 (6–18). Of the children with definite TBM, almost one-third (n=17, 31.5%) had scores <12 (figure 1).

### Radiological investigations

Of 112 patients who underwent chest radiography, 81 (72.3%) had changes suggestive of intrathoracic TB disease, including hilar lymphadenopathy, pulmonary infiltrates, consolidation or cavitation (table 2). In 26 (22.8%) cases miliary infiltrates were identified.

**TABLE 2:** Radiological findings on chest radiography, abdominal ultrasound scan and cranial imaging with computed tomography (CT) or magnetic resonance imaging (MRI)

**Chest radiography**	112
Changes suggestive of active TB^#^	81 (72.3)
Changes suggestive of previous pulmonary TB^¶^	2 (1.8)
No abnormalities detected	29 (25.9)
**Abdominal ultrasound scan**	65
Hepatomegaly	10 (15.4)
Splenomegaly	9 (13.8)
Intra-abdominal granulomas	11 (16.9)
Miliary lesions	4 (6.2)
Enlarged lymph nodes	2 (3.1)
No abnormalities detected	42 (64.6)
**Cranial CT/MRI**	108
Hydrocephalus	53 (49.1)
Basal meningeal enhancement	44 (40.7)
Intracranial tuberculomas	32 (29.6)
Cerebral infarcts	14 (13.0)
No abnormalities detected	18 (16.7)

Data are presented as n or n (%). TB: tuberculosis. ^#^: hilar adenopathy, pulmonary infiltrates, consolidation or cavitation; ^¶^: fibrotic scars or calcification.

65 patients had an abdominal ultrasound scan. In the majority (64.6%), no abnormalities were detected. The most common abnormal findings were hepatomegaly, splenomegaly and intrabdominal granulomas (table 2).

In 108 patients, cranial imaging with computed tomography and/or magnetic resonance imaging was performed. Of those, 90 (83.3%) had one or more abnormal findings consistent with TBM. The most common finding was hydrocephalus, followed by basal meningeal enhancement and intracranial tuberculomas (table 2). In the remaining 18 (16.7%) patients, no significant abnormalities were identified, which included six children with definite TBM.

### Performance of immunological TB tests

TST, QuantiFERON Gold assay and T-SPOT.TB assay (both performed on blood samples) results were available in 92, 53 and 17 patients, respectively. In 10 patients neither TST nor IGRA results were available.

Table 3 summarises the results of 108 patients in whom TST and/or IGRA results were available. Of the 54 children in whom both TST and IGRA results were available only six (11.1%) had concordantly negative TST (at the 10-mm threshold) and IGRA results; five (9.3%) had negative TST and indeterminate QFT results; in the remaining 43 (79.6%), at least one immunological test result was positive.

**TABLE 3:** Summary of tuberculin skin test (TST) and interferon-γ release assay (IGRA) results in patients who had at least one immunological test performed

	**Patients**	**QFT positive**	**QFT negative**	**QFT indeterminate**	**T-SPOT positive**	**T-SPOT negative**	**T-SPOT indeterminate**	**No IGRA^#^**
**TST cut-off 5 mm^¶^**	84							
Positive		18 (21.4)	1 (1.2)	1 (1.2)	8 (9.5)	0	0	24 (28.5)
Negative		9 (10.7)	2 (2.4)	3 (3.6)	4 (4.8)	2 (2.2)	0	12 (14.3)
**TST cut-off 10 mm**	92							
Positive		16 (17.4)	1 (1.1)	1 (1.1)	6 (6.5)	0	0	22 (23.9)
Negative		13 (14.1)	4 (4.3)	5 (5.4)	6 (6.5)	2 (2.2)	0	16 (17.4)
**No TST^+^**	16	9 (56.3)	1 (6.3)	3 (18.8)	2 (12.5)	1 (6.3)	0	

Data are presented as n or n (%). n=108. QFT: QuantiFERON-TB Gold assay; T-SPOT: T-SPOT.TB assay. ^#^: IGRA not performed or result not available; ^¶^: excludes eight patients who were negative at the 10-mm cut-off, but had no quantitative result recorded; ^+^: TST not performed or result not available.

At the 5-mm cut-off the TST had a sensitivity of 61.9% (95% CI 51.2–71.6%), and at the 10-mm cut-off 50.0% (95% CI 40.0–60.0%). The sensitivities of the QFT and the T-SPOT.TB assay were 71.7% (95% CI 58.4–82.1%) and 82.5% (95% CI 58.2–94.6%), respectively. Statistically, there was no significant difference between the sensitivity of the TST at the 5-mm cut-off and the QFT assay (0.27); however, there was a significant difference at the 10-mm cut-off (p=0.0143). Similarly, no significant difference was detected between the TST at 5 mm and the T-SPOT.TB assay (p=0.16), but there was a significant difference at the 10 mm cut-off (p=0.0167). There was no statistically significant difference between the sensitivity of both IGRA assays (p=0.53). The proportion of positive TST results (at the 10-mm cut-off) did not differ significantly between BCG-vaccinated and BCG nonvaccinated children (47.1% *versus* 52.5%; p=0.78); in addition, there was no significant difference between those two subgroups with regards to TST induration size (median 8 mm *versus* 10 mm; p=0.81).

Of the 53 patients with QFT results, nine (17.0%) had an indeterminate test result (95% CI 9.0–29.5%). In the 17 patients with T-SPOT.TB results, there were no indeterminate results. There was no statistical difference between both IGRAs with regards to the proportion of indeterminate *versus* determinate (*i.e.* positive or negative) results (p=0.10). On average children with indeterminate test results were younger (median (IQR) age 2.0 (1.4–3.0) years) than children with determinate results (2.7 (1.0–6.5) years), although this did not reach statistical significance (p=0.22) (supplementary table S2).

### CSF results

**FIGURE 2:**
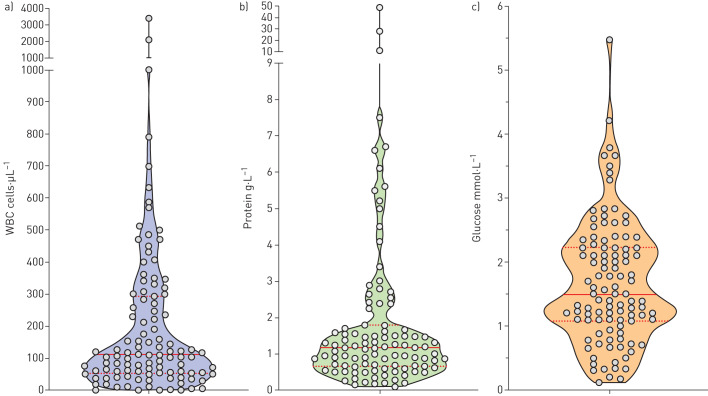
Violin plots of cerebrospinal fluid white blood cell counts (WBC), protein and glucose concentrations at initial presentation. Horizontal lines indicate the medians and interquartile ranges.

The CSF results at initial presentation in the 106 patients in whom those data were available are summarised in figure 2 and supplementary table S3. Only 10 (9.4%) patients had CSF protein concentrations within the normal range (0–0.4 g·L^−1^); in 57 (53.8%) patients, the CSF protein concentration was ≥1.0 g·L^−1^.

### Performance of microbiological tests with CSF

Results of acid-fast stains, mycobacterial culture and *M. tuberculosis* PCR testing on CSF were available in 75, 94 and 69 patients, respectively. Only three (4.0%) cases were positive for acid-fast bacilli (sensitivity 95% CI 0.9–11.6). 47 (50.0%) cases had positive mycobacterial culture results (95% CI 40.1–59.9%), while only 24 (34.8%) were positive on PCR testing (95% CI 22.9–43.7%), although this did not reach statistical significance (p=0.06). Of the 62 cases in whom both mycobacterial culture and PCR had been performed on CSF, 17 were positive in both tests, 15 were positive only in culture, and four positive only in PCR. In this subgroup, performing both tests in parallel achieved greater sensitivity (36 (58.1%) out of 62, 95% CI 45.7–69.5%) than performing either culture (32 (51.6%) out of 62, 95% CI 39.5–63.6%) or PCR alone (21 (33.9%) out of 62, 95% CI 23.3–46.3%), but only the comparison with PCR alone was statistically significant (p=0.59 and p=0.0113, respectively).

### Combining immunological and microbiological tests

**FIGURE 3:**
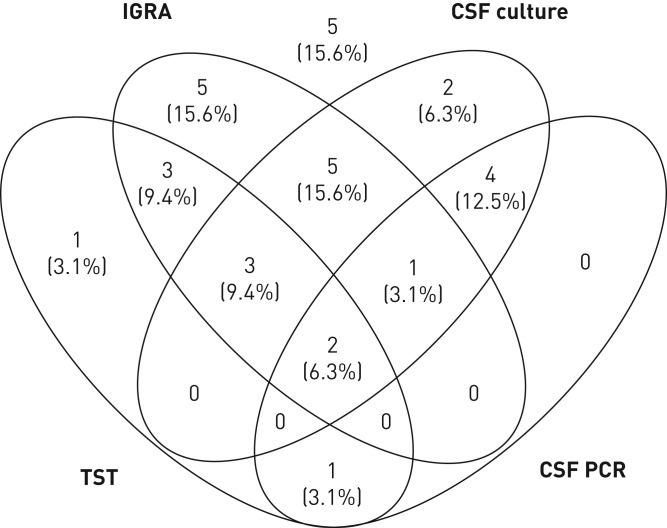
Venn diagram summarising positive tuberculin skin test (TST), interferon-γ release assay (IGRA) and cerebrospinal fluid (CSF) test results (culture and PCR) in the subgroup of patients who had all four tests performed (n=32). In five of those patients (shown above the diagram), all four tests were negative.

Figure 3 summarises the results of the subgroup of patients in whom both immunological and both mycobacterial culture and PCR on CSF had been performed (n=32), showing that TST-positive/IGRA-positive/CSF culture-positive and IGRA-positive only were the most common result constellations, but also that result constellations were very heterogeneous. Only five (15.6%) cases had negative results in all four tests; therefore, the overall sensitivity of all four tests combined was 84.4% (95% CI 67.8%–93.6%). Only one of those five cases had sampling performed at another site, a lymph node biopsy that was culture- and PCR-positive for *M. tuberculosis*.

### Multivariate logistic regression analyses

The results of the multivariate regression analyses for having a positive immunological test result are summarised in table 4. Children with TBM grade 3 were significantly less likely to have a positive TST (at both the 5-mm (OR 0.08, p=0.019) and the 10-mm cut-off (OR 0.06, p=0.023)) than those with TBM grade 1. However, this was not a statistically significant predictor of a positive IGRA result (OR 0.13, p=0.13). Younger children were more likely to have a positive IGRA result (OR 0.7 per year of increasing age, p=0.042). There were no statistically significant predictor variables of an indeterminate IGRA result in multivariate analysis (supplementary table S2).

**TABLE 4:** Multivariate logistic regression analysis for association between positive tuberculin skin test (TST) at the 5-mm and the 10-mm threshold, and positive interferon-γ release assay result (IGRA^+^) as outcome variable, and predictor variables of age, sex, bacille Calmette–Guérin (BCG) vaccination status, tuberculosis meningitis (TBM) staging, definite TBM diagnosis and IGRA type (for IGRA result only)

	**Descriptor**	**Outcome variable (positive result)**	**OR (95% CI)**	**p-value**
**Age years**	Continuous	TST 5 mm	0.89 (0.78–1.02)	0.100
		TST 10 mm	0.90 (0.79–1.02)	0.103
		IGRA^+^	**0.70 (0.50–0.99)**	**0.042**
**Male**	Binary	TST 5 mm	0.43 (0.14–1.29)	0.133
		TST 10 mm	0.61 (0.22–1.66)	0.331
		IGRA^+^	0.66 (0.16–2.69)	0.560
**BCG status**	Binary	TST 5 mm	1.60 (0.33–7.71)	0.560
		TST 10 mm	1.27 (0.29–5.50)	0.751
		IGRA^+^	1.52 (0.13–18.08)	0.740
**TBM staging**	Categorical			
Stage 2 compared to stage 1		TST 5 mm	0.36 (0.11–1.21)	0.100
		TST 10 mm	0.43 (0.15–1.25)	0.121
		IGRA^+^	0.25 (0.03–1.85)	0.180
Stage 3 compared to stage 1		TST 5 mm	**0.08 (0.01–0.65)**	**0.019**
		TST 10 mm	**0.06 (0.00–0.68)**	**0.023**
		IGRA^+^	0.13 (0.01, 1.82)	0.129
**Definite TBM diagnosis**	Binary	TST 5 mm	0.71 (0.24–2.10)	0.540
		TST 10 mm	0.37 (0.13–1.03)	0.056
		IGRA^+^	0.82 (0.19–3.64)	0.798
**Type of IGRA assay**	Binary			
T-SPOT.TB compared to QFT		IGRA^+^	22.15 (0.79–623.49)	0.069

Indeterminate IGRA results were classified as negative for this analysis. Bold type represents p<0.05. T-SPOT: T-SPOT.TB assay; QFT: QuantiFERON-TB Gold assay.

### Detection of *M. tuberculosis* at other sites

In 56 (47.5%) patients *M. tuberculosis* was detected in at least one clinical sample other than CSF (table 5). Of those, 49 (87.5%) were culture-positive and 27 (48.2%) were PCR-positive; 20 (35.7%) were positive in both culture and PCR. Among the 64 cases with possible and probable TBM (*i.e.* in whom *M. tuberculosis* was not identified in the CSF), *M. tuberculosis* was identified by culture and/or PCR at another site in 30 (46.9%) cases, securing a microbiological diagnosis in those patients.

**TABLE 5:** Summary of microbiological test results of samples other than cerebrospinal fluid

	**Patients**	**Positive for AFB**	**Positive in culture**	**Positive in PCR**
**Sputum**	8	1 (12.5)	6 (75.0)	0
**Gastric aspirates**	42	10 (23.8)	37 (88.1)	18 (42.9)
**Nasopharyngeal aspirate**	5	1 (20.0)	4 (80)	3 (60.0)
**Bronchoalveolar lavage fluid**	7	3 (42.9)	6 (85.7)	3 (42.9)
**Lymph node material**	4	0	3 (75.0)	3 (75.0)

Data are presented as n or n (%). AFB: acid-fast bacilli.

## Discussion

To our knowledge, this is the largest multicentre study on TBM in children from a low TB incidence setting to date, facilitated by inclusion of a large number of participating centres across Europe via a well-established collaborative paediatric TB research network.

That this study was conducted in a low TB incidence setting is relevant for the interpretation of its findings. Almost a third (33.1%) of the cases had BMRC TBM grade 1 disease, while only 9.3% had grade 3 disease, contrasting with reports from high TB incidence countries, where the vast majority of patients have grade 2 or grade 3 disease at presentation [13]. This indicates that in the European setting there is a tendency for patients with TBM to present earlier and with less severe disease.

In addition, our cohort differs from most cohorts of children with TBM reported from high TB prevalence countries with regards to the proportion of cases that are microbiologically confirmed. Almost half (45.8%) of the cases in our cohort were confirmed, contrasting with studies from high prevalence settings in which fewer than a quarter were confirmed cases [14, 19, 20]. This may be the result of recall bias and preferential reporting of confirmed cases in our study, or alternatively may reflect the greater availability of diagnostic tests at the centres that participated in this study compared to lower resource settings. The latter hypothesis is supported by the fact that paediatric studies in high TB prevalence settings with access to molecular diagnostics have reported similar proportions of microbiologically confirmed cases [4, 21].

The performance of the UTRCD scoring system, which is based on expert consensus [3], was suboptimal in our cohort. Almost one-third (31.5%) of cases with microbiologically confirmed TBM had scores <12, which in the absence of a positive microbiological result would categorise those patients as “possible TBM”. It is possible that the comparatively poor performance relates to the tendency for European patients to present earlier (and therefore with fewer features and consequently lower scores) than patients in high TB prevalence settings. However, in some patients, certain data required for scoring (*e.g.* symptom duration) were not recorded, potentially resulting in the UTRCD scores of those patients being skewed towards lower values. However, importantly, this scoring system was developed for research purposes, and not for clinical decision-making.

Radiological changes suggestive of intrathoracic TB disease were present in almost three-quarters (72.3%) of the cases. This highlights that patients with suspected TBM should routinely undergo chest imaging, as this is likely to provide useful information aiding diagnosis. However, our data contrast with data from other studies that reported chest radiography changes in considerably lower proportions of children with TBM, typically 40–60% [19, 22, 23]. The observation that a high proportion (83.3%) of children in our cohort had abnormal findings on cranial imaging, including hydrocephalus, basal meningeal enhancement and intracranial tuberculomas, is consistent with previous reports [19, 20, 22].

Our data highlight that all existing immunological and microbiological TB tests have suboptimal sensitivity in children with TBM. TST, QFT assays and T-SPOT.TB assays had sensitivities of ∼80% or below, indicating that approximately one in five children with TBM have a false-negative result when a single immunological test is performed, irrespective of which test is used. Several recent studies, including in patients with TBM, have investigated novel immune-based TB biomarkers in blood that have the potential to improve the diagnosis of TB in children, but additional studies will be required to confirm their findings [24–26]. Interestingly, our multivariate logistic regression analyses indicate that false-negative TST results were more common in children with more severe TBM. Furthermore, we found that a large proportion of patients had discordant TST and IGRA results, in accordance with observations reported by studies in children with pulmonary TB [24, 27, 28].

It was striking that a substantial proportion of children had indeterminate IGRA test results. Among children who had undergone testing with QFT assays, 17.0% had an indeterminate result, which is considerably higher than in most studies that investigated the performance of QFT assays in children with pulmonary TB [24, 27, 29, 30]. Interestingly, the association between tuberculous central nervous system (CNS) disease and indeterminate IGRA results was also observed in a Californian study, which only included 17 confirmed cases with CNS disease [11]. Although the basis for these observations remains uncertain, it is tempting to hypothesise that age is a contributing factor, considering that the median age of our cohort was 2.6 years, but we did not detect an association between age and indeterminate test results in multivariate logistic regression analyses. Nevertheless, several published studies, including our own, have shown that young age is associated with indeterminate IGRA results [31–33]. Alternatively, there may be immunological differences between children with TBM and those with pulmonary TB, resulting in impaired IGRA performance in the former.

In accordance with published data our results show that acid-fast bacilli stain microscopy has very poor sensitivity in children with TBM [7, 14, 22]. Mycobacterial culture performed on CSF was the microbiological test with the highest sensitivity, but still only positive in half of the cases. PCR for *M. tuberculosis* performed on CSF had even lower sensitivity, producing a positive result in only approximately one in three cases. However, the fact that a variety of in-house and commercial PCR assays were used at different healthcare institutions limit the interpretation of this finding. A recent study that included 23 HIV-infected adults with TBM found that the recently released Xpert MTB/RIF Ultra assay had higher sensitivity than both the previous generation Xpert MTB/RIF assay and culture (sensitivity 70% *versus* 43% and 43%, respectively) [34], suggesting that some commercial PCR-based assays may perform as well as culture or potentially even have superior sensitivity in TBM. However, larger prospective studies are needed to confirm those findings. Our data show that performing both culture and PCR on CSF in parallel increases the diagnostic yield, in concordance with observations in a paediatric study from South Africa [21]. A recent publication raises hopes that metagenomic next-generation sequencing of CSF could potentially improve the diagnosis of CNS infections with organisms that are difficult to detect with existing microbiological methods [35].

In addition, our data show that in the large majority of children with TBM at least one test produces a positive result if TST, IGRA, CSF culture and CSF PCR are performed in parallel, as only 15.6% of the cases showed false-negative results in all four tests. In the paediatric setting immunological tests are often used as adjunctive tests in suspected TB disease [8], a practice that is supported by our findings. Although positive immunological tests do not confirm TB disease, in a child with compatible clinical and radiological findings they lend substantial support to a putative diagnosis of TBM.

In a substantial number of children in this cohort *M. tuberculosis* was detected in samples other than CSF, helping to secure a microbiological diagnosis. Sputum, gastric aspirates and bronchoalveolar lavage fluid samples all had high yields, universally with a positive detection rate of ≥75%. However, considering that decisions to obtain those samples were made by clinicians managing the patients, rather than according to a standardised study protocol, it is probable that these samples were preferentially obtained in a selected group of patients who had chest radiography changes or respiratory symptoms. Nevertheless, our data highlight that testing respiratory/gastric samples should be undertaken routinely in children with suspected TBM, as *M. tuberculosis* can often not be detected in CSF, precluding microbiological confirmation and susceptibility testing.

As with all retrospective studies, a key limitation is that some data were missing due to incomplete documentation. A larger number of patients had TSTs performed, but only categorical rather than quantitative data were documented, resulting in those patients having to be excluded from some of the analyses. Additionally, only a small number of participating centres were using T-SPOT.TB assays; consequently, the data on the performance of this test were limited. No cases had IGRAs performed on CSF; however, a recent meta-analysis has shown that performing IGRAs on CSF rather than blood does not result in a higher diagnostic yield [12]. Finally, in common with all retrospective studies, there is a risk of recall bias and preferential reporting.

In conclusion, our data show that in the European setting children with TBM tend to present earlier and with less severe disease than in high TB incidence settings. A large proportion of children with TBM have coexisting intrathoracic TB disease, and consequently chest imaging and collection of respiratory or gastric samples should be considered in all patients with suspected TBM. Both immunological (TST and IGRAs) and microbiological TB tests have suboptimal sensitivity in children with TBM. Performing both TST and IGRA in parallel with microbiological testing of CSF by culture and PCR results in a substantial increase in the proportion of children who have evidence of TB infection, which should therefore be the standard approach in healthcare settings with sufficient resources to perform those tests.

## Supplementary material

10.1183/13993003.02004-2019.Supp1 **Please note:** supplementary material is not edited by the Editorial Office, and is uploaded as it has been supplied by the author.
Supplementary material ERJ-02004-2019.Supplement

## Shareable PDF

10.1183/13993003.02004-2019.Shareable1 This one-page PDF can be shared freely online.
Shareable PDF ERJ-02004-2019.Shareable

## Supplementary Material

ERJ-02004-2019.Shareable.pdf

ERJ-02004-2019.Supplement.pdf
