# Supplementary material for: Performance of immune-based and microbiological tests in children with tuberculosis meningitis in Europe: a multicentre Paediatric Tuberculosis Network European Trials Group (ptbnet) study
Source: Eur Respir J. 2020 Jul 2;56(1):1902004. doi: 10.1183/13993003.02004-2019 (PMC7330130; doi:10.1183/13993003.02004-2019)

Performance of immune-based and microbiological tests in children with TB meningitis in Europe – a multi-center Paediatric Tuberculosis Network European Trials Group (ptbnet) study

Robindra Basu Roy [1*], Stephanie Thee [2*], Daniel Blázquez-Gamero [3], Lola Falcón-Neyra [4], Olaf Neth [4], Antoni Noguera-Julian [5,6,7,8], Cristina Lillo [3], Luisa Galli [9,10], Elisabetta Venturini [9,10], Danilo Buonsenso [11], Florian Götzinger [12], Nuria Martinez-Alier [13], Svetlana Velizarova [14], Folke Brinkmann [15], Steven B Welch [16], Maria Tsolia [17], Begoña Santiago-Garcia [18], Renate Krüger [2], Marc Tebruegge, [13,19,20]

on behalf of the ptbnet TB Meningitis Study Group **

1. Clinical Research Department, London School of Hygiene & Tropical Medicine, London, UK.
2. Department of Pediatric Pneumology, Immunology and Intensive Care, Charité – Universitätsmedizin Berlin, Berlin, Germany.
3. Paediatric Infectious Diseases Unit, Hospital Universitario 12 de Octubre, Universidad Complutense de Madrid, Instituto de Investigación Hospital Universitario 12 de Octubre (imas12), RITIP, Madrid, Spain.
4. Paediatric Infectious Diseases, Rheumatology and Immunology Unit, Hospital Universitario Virgen del Rocío, Institut of Biomedicine of Seville (IBIS), Spain.
5. Malalties Infeccioses i Resposta Inflamatòria Sistèmica en Pediatria, Institut de Recerca Pediàtrica; Hospital Sant Joan de Déu, Barcelona, Spain.
6. Departament de Pediatria, Universitat de Barcelona, Barcelona, Spain.
7. CIBER de Epidemiología y Salud Pública, CIBERESP, Madrid, Spain.
8. Red de Investigación Translacional en Infectología Pediátrica, RITIP, Madrid, Spain.
9. Department of Health Sciences, University of Florence, Florence, Italy.
10. Paediatric Infectious Disease Unit, Meyer Children's University Hospital, Florence, Italy.
11. Department of Woman and Child Health and Public Health, Fondazione Policlinico Universitario A. Gemelli IRCCS, Rome, Italy.
12. Department of Paediatrics and Adolescent Medicine, Wilhelminenspital, Vienna, Austria.

13. Department of Paediatric Infectious Diseases & Immunology, Evelina London Children’s Hospital, Guy’s and St. Thomas’ NHS Foundation Trust, London, UK.
14. Department of Pulmonary Diseases, Medical University, Hospital for Lung Diseases ‘St. Sofia’, Sofia, Bulgaria.
15. Department of Paediatric Pulmonology, Ruhr University Bochum, Bochum, Germany.
16. Birmingham Chest Clinic and Heartlands Hospital, University Hospitals Birmingham, Birmingham, UK.
17. Second Department or Paediatrics, National and Kapodistrian University of Athens, School of Medicine, P. and A. Kyriakou Children’s Hospital, Athens, Greece.
18. Department of Paediatric Infectious Diseases, Hospital General Universitario Gregorio Marañón, Madrid, Spain.
19. Department of Paediatrics, Royal Children’s Hospital Melbourne, University of Melbourne, Melbourne, Australia.
20. Department of Infection, Immunity & Inflammation, UCL Great Ormond Street Institute of Child Health, University College London, London, UK.

* joint first authors

**** ptbnet TB Meningitis Study Group Collaborators:**
Matthias Bogyi, Wilhelminenspital Vienna, Austria; Carlotta Montagnani, Anna Meyer Children's University Hospital Florence, Italy; Laura Lancella, Bambino Gesù Children Hospital Rome, Italy; Eeva Salo, University of Helsinki Children's Hospital, Finland; Angeliki Syngelou, 2nd Dept of Paediatrics, National & Kapodistrian University of Athens, P. & A. Kyriakou Children’s Hospital, Greece; Uros Krivec, University Children's Hospital Ljubljana, Slovenia; Andrea Martín Nalda and Antoni Soriano-Arandes, Hospital Vall d´Hebron Barcelona, Spain; Irene Rivero, Hospital Clínico Universitario de Santiago de Compostela, Spain; Marta Benavides Nieto, Hospital Infantil Virgen del Rocío Sevilla, Spain; Mercedes Bueno, Hospital Universitario Fundación Alcorcón Madrid, Spain; Teresa del Rosal, Hospital Infantil La Paz Madrid, Spain; Luis Mayol and Borja Guarch, Hospital Universitari Dr. Josep Trueta Girona, Spain; Jose Antonio Couceiro, Complejo Hospitalario de Pontevedra, Spain; Carmelo Guerrero Laleona, Hospital Miguel Servet Zaragoza, Spain; Rutger Bennet, Astrid Lindgren Children's Hospital Stockholm, Sweden; Karsten Kötz,

Queen Silvia Children's Hospital Gothenburg, Sweden; Brittany Raffa, Evelina London Children's Hospital, UK; Fiona Shackley, Sheffield Children's Hospital, UK.

Corresponding author:
Dr Marc Tebruegge, Department of Infection, Immunity and Inflammation,
UCL Great Ormond Street Institute of Child Health, University College London
30 Guildford Street, London WC1N 1EH, United Kingdom.
Email: m.tebruegge@ucl.ac.uk

Supplementary Table S1. Uniform TB Meningitis Research Case Definition scoring system (adapted from Marais et al. (3))

	**Diagnostic score**
**Clinical criteria**	(Maximum category score=6)
Symptom duration of more than 5 days	4
Systemic symptoms suggestive of TB (one or more of the following): weight loss (or poor weight gain in children), night sweats, or persistent cough for more than 2 weeks	2
History of recent (within past year) close contact with an individual with pulmonary TB or a positive TST or IGRA (only in children <10 years of age)	2
Focal neurological deficit (excluding cranial nerve palsies)	1
Cranial nerve palsy	1
Altered consciousness	1
**CSF criteria**	(Maximum category score=4)
Clear appearance	1
Cells: 10–500 per µl	1
Lymphocytic predominance (>50%)	1
Protein concentration greater than 1 g/L	1
CSF to plasma glucose ratio of less than 50% or an absolute CSF glucose concentration less than 2.2mmol/L	1
**Cerebral imaging criteria**	(Maximum category score=6)
Hydrocephalus	1
Basal meningeal enhancement	2
Tuberculoma	2
Infarct	1
Pre-contrast basal hyperdensity	2
**Evidence of TB elsewhere**	(Maximum category score=4)
Chest radiograph suggestive of active TB: signs of TB=2; miliary TB=4	2/4
CT / MRI / ultrasound evidence for TB outside the CNS	2
AFB identified or *Mycobacterium tuberculosis* cultured from another source—ie, sputum, lymph node, gastric washing, urine, blood culture	4
Positive commercial *Mycobacterium tuberculosis* NAAT from extra-neural specimen	4
**Exclusion of alternative diagnoses**	

Abbreviations: AFB = acid-fast bacilli; CNS = central nervous system; CSF = cerebrospinal fluid; CT = computed tomography; MRI = magnetic resonance imaging; NAAT = nucleic acid amplification test; TB = tuberculosis; TST = tuberculin skin test

Supplementary Table S2. Multivariate logistic regression analysis for association between indeterminate interferon-gamma release assay result as outcome variable, and predictor variables of age, sex, BCG vaccination status, TB meningitis staging and classification.

**Predictor variable**	**Descriptor**		**Odds Ratio (95% CI)**	**P value**
Age (years)	Continuous		0.93<br>(0.62, 1.40)	0.73
Male	Binary		4.21<br>(0.67, 26.37)	0.13
BCG status	Binary		1.81<br>(0.09, 38.26)	0.70
TBM staging	Categorical	(stage 2 compared to stage 1)	2.33<br>(0.23, 23.68)	0.47
		(stage 3 compared to stage 1)	13.10<br>(0.62, 278.27)	0.10
Definite TBM diagnosis	Binary		1.58<br>(0.29, 8.63)	0.6
Type of IGRA assay*	Binary	T-SPOT.TB compared to QFT	-	-

*Type of IGRA assay omitted from model as no indeterminate IGRA results amongst 17 T-SPOT.TB assays.

Supplementary Table S3. Cerebrospinal fluid laboratory results at initial presentation in patients in whom those data were available (n=106).

	**Median**	**IQR**
**White blood cell count**	110 / µL	55 – 296 / µL
**Neutrophil percentage**	12%	5 – 30%
**Lymphocyte percentage**	82%	64 – 95%
**Protein concentration**	1.18 g/L	0.67 – 1.79 g/L
**Glucose concentration**	1.50 mmol/L	1.08 – 2.22 mmol/L

Abbreviations: IQR: interquartile range

Supplementary Figure S1: Distribution of categorical interferon-gamma release assay results (QuantiFERON-TB and T-SPOT.TB assays) according to age.

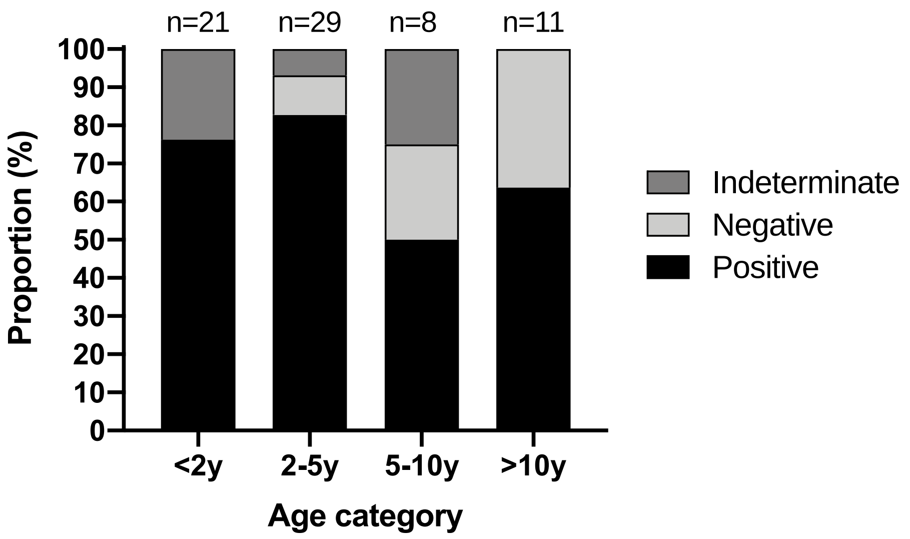

Supplement: Supplementary file 1 [file ERJ-02004-2019.Supplement.pdf]
