# Supplementary material for: Performance of immune-based and microbiological tests in children with tuberculosis meningitis in Europe: a multicentre Paediatric Tuberculosis Network European Trials Group (ptbnet) study
Source: Eur Respir J. 2020 Jul 2;56(1):1902004. doi: 10.1183/13993003.02004-2019 (PMC7330130; doi:10.1183/13993003.02004-2019)
Supplement: Supplementary file 2 [file ERJ-02004-2019.Shareable.pdf]

SHAREABLE PDF

Performance of immune-based and microbiological tests in children with tuberculosis meningitis in Europe: a multicentre Paediatric Tuberculosis Network European Trials Group (ptbnet) study

Robindra Basu Roy[1,21], Stephanie Thee[2,21], Daniel Blázquez-Gamero[3], Lola Falcón-Neyra[4], Olaf Neth[4], Antoni Noguera-Julian[5,6,7,8], Cristina Lillo[3], Luisa Galli[9,10], Elisabetta Venturini[9,10], Danilo Buonsenso[11], Florian Götzinger[12], Nuria Martinez-Alier[13], Svetlana Velizarova[14], Folke Brinkmann[15], Steven B. Welch[16], Maria Tsolia[17], Begoña Santiago-Garcia[18], Renate Krüger[2] and Marc Tebruegge[13,19,20], on behalf of the ptbnet TB Meningitis Study Group[22]

Affiliations: [1]Clinical Research Dept, London School of Hygiene and Tropical Medicine, London, UK. [2]Dept of Pediatric Pneumology, Immunology and Intensive Care, Charité–Universitätsmedizin Berlin, Berlin, Germany. [3]Paediatric Infectious Diseases Unit, Hospital Universitario 12 de Octubre, Universidad Complutense de Madrid, Instituto de Investigación Hospital Universitario 12 de Octubre (imas12), RITIP, Madrid, Spain. [4]Paediatric Infectious Diseases, Rheumatology and Immunology Unit, Hospital Universitario Virgen del Rocío, Institute of Biomedicine, Seville, Spain. [5]Malalties Infeccioses i Resposta Inflamatòria Sistèmica en Pediatria, Institut de Recerca Pediàtrica; Hospital Sant Joan de Déu, Barcelona, Spain. [6]Departament de Pediatria, Universitat de Barcelona, Barcelona, Spain. [7]CIBER de Epidemiología y Salud Pública, CIBERESP, Madrid, Spain. [8]Red de Investigación Translacional en Infectología Pediátrica, RITIP, Madrid, Spain. [9]Dept of Health Sciences, University of Florence, Florence, Italy. [10]Paediatric Infectious Disease Unit, Meyer Children's University Hospital, Florence, Italy. [11]Dept of Woman and Child Health and Public Health, Fondazione Policlinico Universitario A. Gemelli IRCCS, Rome, Italy. [12]Dept of Paediatrics and Adolescent Medicine, Wilhelminenspital, Vienna, Austria. [13]Dept of Paediatric Infectious Diseases and Immunology, Evelina London Children's Hospital, Guy's and St. Thomas' NHS Foundation Trust, London, UK. [14]Dept of Pulmonary Diseases, Medical University, Hospital for Lung Diseases 'St. Sofia', Sofia, Bulgaria. [15]Dept of Paediatric Pulmonology, Ruhr University Bochum, Bochum, Germany. [16]Birmingham Chest Clinic and Heartlands Hospital, University Hospitals Birmingham, Birmingham, UK. [17]Second Dept of Paediatrics, National and Kapodistrian University of Athens, School of Medicine, P. and A. Kyriakou Children's Hospital, Athens, Greece. [18]Dept of Paediatric Infectious Diseases, Hospital General Universitario Gregorio Marañón, Madrid, Spain. [19]Dept of Paediatrics, Royal Children's Hospital Melbourne, University of Melbourne, Melbourne, Australia. [20]Dept of Infection, Immunity and Inflammation, UCL Great Ormond Street Institute of Child Health, University College London, London, UK. [21]Joint first authors. [22]A list of the tbnet TB Meningitis Study Group Collaborators can be found in the acknowledgements section.

Correspondence: Marc Tebruegge, Dept of Infection, Immunity and Inflammation, UCL Great Ormond Street Institute of Child Health, University College London, 30 Guildford Street, London WC1N 1EH, UK. E-mail: m.tebruegge@ucl.ac.uk

@ERSpublications
All existing immunological and microbiological TB tests have suboptimal sensitivity in children with TBM. Combining immune-based tests with CSF culture and PCR results in far higher positive diagnostic yields, and should therefore be standard practice. http://bit.ly/2TSAArl

Cite this article as: Basu Roy R, Thee S, Blázquez-Gamero D, *et al*. Performance of immune-based and microbiological tests in children with tuberculosis meningitis in Europe: a multicentre Paediatric Tuberculosis Network European Trials Group (ptbnet) study. *Eur Respir J* 2020; 56: 1902004 [https://doi.org/10.1183/13993003.02004-2019].

Copyright ©ERS 2020. This version is distributed under the terms of the Creative Commons Attribution Licence 4.0.

This single-page version can be shared freely online.

ABSTRACT

Introduction: Tuberculous meningitis (TBM) is often diagnostically challenging. Only limited data exist on the performance of interferon-γ release assays (IGRA) and molecular assays in children with TBM in routine clinical practice, particularly in the European setting.

Methods: Multicentre, retrospective study involving 27 healthcare institutions providing care for children with tuberculosis (TB) in nine European countries.

Results: Of 118 children included, 54 (45.8%) had definite, 38 (32.2%) probable and 26 (22.0%) possible TBM; 39 (33.1%) had TBM grade 1, 68 (57.6%) grade 2 and 11 (9.3%) grade 3. Of 108 patients who underwent cranial imaging 90 (83.3%) had at least one abnormal finding consistent with TBM. At the 5-mm cut-off the tuberculin skin test had a sensitivity of 61.9% (95% CI 51.2–71.6%) and at the 10-mm cut-off 50.0% (95% CI 40.0–60.0%). The test sensitivities of QuantiFERON-TB and T-SPOT.TB assays were 71.7% (95% CI 58.4–82.1%) and 82.5% (95% CI 58.2–94.6%), respectively ($p=0.53$). Indeterminate results were common, occurring in 17.0% of QuantiFERON-TB assays performed. Cerebrospinal fluid (CSF) cultures were positive in 50.0% (95% CI 40.1–59.9%) of cases, and CSF PCR in 34.8% (95% CI 22.9–43.7%). In the subgroup of children who underwent tuberculin skin test, IGRA, CSF culture and CSF PCR simultaneously, 84.4% had at least one positive test result (95% CI 67.8%–93.6%).

Conclusions: Existing immunological and microbiological TB tests have suboptimal sensitivity in children with TBM, with each test producing false-negative results in a substantial proportion of patients. Combining immune-based tests with CSF culture and CSF PCR results in considerably higher positive diagnostic yields, and should therefore be standard clinical practice in high-resource settings.
